# Correspondence

**Published:** 1888-08

**Authors:** 


Gentlemen:—Your reply to my query on page 385, July, 1888, is not as lucid a one as I had expected. If you had cured an aggravated case of "*Rhus* poisoning" with some potency of Rhus, would you—I beg your pardon—would *I* be justified in administering potentized Morphia, say the CM or DM, if said drug was "found to be well indicated by the symptoms," notwithstanding the fact that such symptoms were produced by poisoning from an overdose of the same poison (Morphia)? * * * *

